# Caffeine as a tool for investigating the integration of Cdc25 phosphorylation, activity and ubiquitin-dependent degradation in *Schizosaccharomyces pombe*

**DOI:** 10.1186/s13008-020-00066-1

**Published:** 2020-06-29

**Authors:** John P. Alao, Per Sunnerhagen

**Affiliations:** 1 School of Health, Sports and Bioscience, University of East London, Stratford Campus, London, E15 4LZ UK; 2 Department of Chemistry and Molecular Biology, University of Gothenburg, Box 462, Gothenburg, SE- 405 30 Sweden

**Keywords:** *Schizosaccharomyces pombe*, Fission yeast, Ubiquitin, 26S proteasome, Cdc25, Caffeine, Cell cycle, DNA damage checkpoints, Phosphorylation

## Abstract

The evolutionarily conserved Cdc25 phosphatase is an essential protein that removes inhibitory phosphorylation moieties on the mitotic regulator Cdc2. Together with the Wee1 kinase, a negative regulator of Cdc2 activity, Cdc25 is thus a central regulator of cell cycle progression in *Schizosaccharomyces pombe*. The expression and activity of Cdc25 is dependent on the activity of the Target of Rapamycin Complex 1 (TORC1). TORC1 inhibition leads to the activation of Cdc25 and repression of Wee1, leading to advanced entry into mitosis. Withdrawal of nitrogen leads to rapid Cdc25 degradation via the ubiquitin- dependent degradation pathway by the Pub1 E3- ligase. Caffeine is believed to mediate the override of DNA damage checkpoint signalling, by inhibiting the activity of the ataxia telangiectasia mutated (ATM)/Rad3 homologues. This model remains controversial, as TORC1 appears to be the preferred target of caffeine in vivo. Recent studies suggest that caffeine induces DNA damage checkpoint override by inducing the nuclear accumulation of Cdc25 in *S. pombe*. Caffeine may thus modulate Cdc25 activity and stability via inhibition of TORC1. A clearer understanding of the mechanisms by which caffeine stabilises Cdc25, may provide novel insights into how TORC1 and DNA damage signalling is integrated.

## Background

**Fig. 1:**
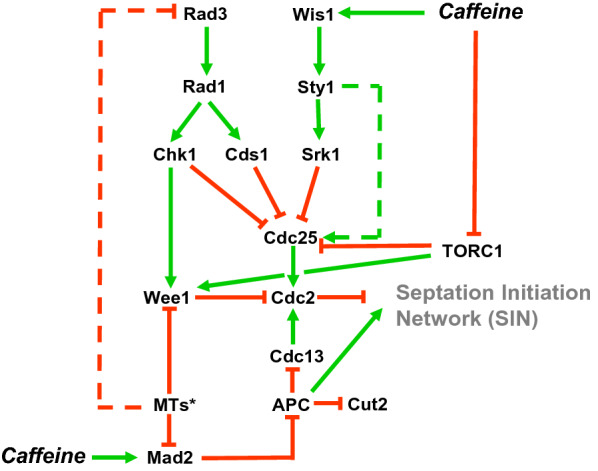
Effect of caffeine on Cdc25 regulation in *S. pombe*. Cdc2- Cdc13 is regulated by Cdc25 and Wee1. Suppression of Cdc2 activity by the anaphase promoting complex (APC), facilitates mitotic exit and activation of the septation initiation network (SIN). Caffeine was initially thought to inhibit Rad3 activity resulting in DNA damage checkpoint override. More recent studies have identified the TORC1 complex as the major target of caffeine in vivo. TORC1 delays mitosis by negatively regulating Cdc25 and activating Wee1. TORC1 inhibition advances the timing of mitosis suggesting caffeine can modulate cell cycle progression by inhibiting this complex. Caffeine activates the Sty1 regulated environmental stress response (ESR) pathway, leading to partial Cdc25 inhibition by Srk1. Depending on the degree of activation, Sty1 can also modulate Cdc25 activity to advance mitosis. The Mad2 spindle checkpoint protein is involved in the regulation of the DNA replication checkpoint. Caffeine’s effect on cell cycle progression is partially inhibited by Mad2. *MTs (Microtubules). Green arrows indicate target activation. Red lines indicate inhibitory signalling

The tightly regulated timing of mitosis in *S. pombe* occurs via the reciprocal activities of Cdc25 and Wee1 on Cdc2 inhibitory phosphorylation. Wee1 negatively regulates Cdc2 by phosphorylation of tyrosine residue 15 (Tyr15), and this is counteracted by the phosphatase activity of Cdc25 [1–3]. Cells must advance or delay mitosis under nutrient stress or genotoxic/environmental stress conditions respectively, several signalling pathways converge on the regulation of the Cdc25- Wee1 dual switch to effect accelerated entry into mitosis or a “double- lock” checkpoint mechanism. These pathways include the Target of Rapamycin Complex 1 (TORC1), the DNA damage response (DDR) and the environmental stress response (ESR) pathways [3–7] (Fig. 1).

The methylxanthine caffeine is among the most widely used neuroactive substances in the world [8–11]. Caffeine exerts various effects on cellular and organismal physiology and is known to inhibit several members of the phosphatidylinositol 3 kinase-like kinase (PIKK) family including ataxia telangiectasia mutated (ATM) and ataxia telangiectasia and rad related (ATR) kinase homologue Rad3 and TORC1 in vitro [10, 12–14]. Early studies suggested that caffeine overrides DNA damage checkpoint signalling, by inhibiting Rad3 and its homologues but this view remains controversial [12, 15]. Interestingly, TORC1 appears to be the major cellular target of caffeine in vivo [15–17]. The Tor2-containing TORC1 complex is a negative regulator of Cdc25 activity that determines the timing of mitosis in response to nutrient availability [18, 19]. We and others have previously demonstrated that caffeine induces Cdc25 accumulation in mammalian and *S. pombe* cells [20, 21]. The mechanisms by which caffeine stabilises Cdc25 in *S. pombe* remain unclear, but do not result from increased *cdc25*^+^ mRNA expression. Furthermore, Cdc25 expression was required for caffeine- mediated DNA damage checkpoint override in *S. pombe*. Intriguingly the effect of caffeine on cell progression under normal growth conditions mimics that of TORC1 inhibition [21]. Caffeine may thus modulate the activity of several pathways that converge on the regulation of Cdc25. In fact, caffeine clearly activates the ESR pathway [21, 22]. One interesting question concerns how, the regulation of Cdc25 activity, phosphorylation and ubiquitin- dependent degradation of Cdc25 activity is integrated [23–25]. Given that cross talk occurs between the TORC1, DDR and ESR pathways [26–28], understanding how caffeine modulates Cdc25 activity and stability in *S. pombe* may shed further light on how these pathways interact [4, 6, 21, 29]. Although the co-regulation of Cdc25 and Wee1 is crucial for the proper timing of mitosis or cell cycle arrest and is effected via the same pathways [30]; this review will focus mainly on Cdc25 regulation for simplicity.

## Main text

### Cell cycle dependent regulation of Cdc25 activity, phosphorylation and ubiquitin- dependent degradation by the 26S proteasome

Cdc25 levels oscillate during cell cycle progression in a manner similar to cyclins, rising steadily throughout the cell cycle, before becoming hyper- phosphorylated and degraded during mitosis [1, 2, 23, 31]. Expression of Cdc25 appears to be dependent on TORC1 activity, as nutrient deprivation leads to a rapid loss of expression [1, 2]. In the absence of a nitrogen source, *cdc25*^+^ mRNA translation ceases and the protein is rapidly degraded via the ubiquitin- dependent 26S proteasome pathway [32–34]. Wee1- mediated phosphorylation of Cdc2 tyrosine residues negatively regulates the activity of Cdc2- Cdc13 Maturation Promoting Factor (MPF). Cdc25 removes inhibitory phosphorylation on the Cdc2, leading to an autocatalytic positive feed- back loop, repression of Wee1 activity and full Cdc25 activation [1, 31, 35].

The HECT- type ubiquitin ligase Pub1 targets Cdc25 for ubiquitin- dependent 26S proteasome degradation in *S. pombe*. Deletion of *pub1*^+^ raises Cdc25 levels and renders cells resistant to Wee1 activity. Furthermore, the cyclic expression pattern of Cdc25 appears deregulated in *pub1*Δ mutants [32, 34]. Of note is that *pub1*Δ mutants exhibit several phenotypes, suggesting additional Pub1 substrates. Interestingly, Pub1 also controls the ubiquitin- dependent regulation of amino acid uptake potentially linking nutrient absorption to Cdc25 and cell division via Sty1 and TORC1 [36–38]. The Anaphase- Promoting Complex (APC) may also facilitate the degradation of Cdc25 at mitosis [39, 40].

Cdc25 is a highly unstable protein with a relatively short half- life [2, 34]. Cdc25 levels oscillate through the cell cycle, peaking at mitosis and then rapidly decline just prior to cytokinesis [1, 2, 23, 34]. Recent studies by Lucena et al. [23] reveal that Cdc25 in *S. pombe* becomes highly phosphorylated in G2, becomes dephosphorylated and then hyper- phosphorylated between mitosis and cytokinesis. Cdc25 levels then decline as the cells proceed through mitosis. Phosphorylation of Cdc25 during normal cell cycle progression is dependent on Cdc2 phosphorylation sites [23, 41]. The decrease in both phosphorylated and total Cdc25 levels was strongly associated with a rise in cyclin Cdc13 levels [23]. Dephosphorylation of Cdc25 at mitosis is regulated by the protein phosphatase 2A and its regulatory subunit Pab1 (PP2A^Pab1^). In mutants lacking *pab1*^+^, Cdc25 remains hyperphosphorylated throughout the cell cycle and the timing of mitosis exceeds that of wild type cells. The degradation of Cdc25 still occurs in strains expressing mutant isoforms lacking Cdc2 phosphorylation sites, as well as in *pab1*Δ mutants. In addition, the relative abundance of Cdc25 during the cell cycle in *pab1*Δ mutants is unaffected [23]. We have also detected a Cdc25 expression negative- feedback loop in *S. pombe* [21]. Similarly, Clp1 phosphatase activity enhances the rate of Pub1-mediated Cdc25 degradation and timing of mitosis [34, 39, 42]. In *clp1*Δ mutants, Cdc25 remains phosphorylated throughout the cell cycle and the cell cycle is lengthened relative to wild type cells. Levels of Cdc25 are also elevated relative to wild type cells in *clp1*Δ mutants [23, 34]. Clp1 also cooperates with the Pub1 and APC E3- ligases to facilitate the rapid degradation of Cdc25 at mitosis [34, 39, 40, 42]. PP2A^Pab1^ and Clp1 phosphatase activity and Cdc25 degradation are thus important for regulating the timing of mitosis. In fact, high Cdc2 activity delays the timing of mitosis in *S. pombe* by inhibiting the septation initiation network (SIN) [34, 39, 40]. Hence, the link between Cdc25 phosphorylation, activity and degradation remains unclear (discussed further below) [24].

Importantly, under normal cell cycle conditions TORC1 inhibits the Greatwall kinase phosphorylates Endosulfine, which is a potent inhibitor of PP2A^Pab1^ phosphatase activity. When nitrogen is withdrawn or TORC1 is chemically inhibited, PP2A^Pab1^ is indirectly inhibited, Cdc25 becomes hyperphosphorylated and entry into mitosis in these cells is advanced. This activity also links the Sty1 regulated environmental stress response pathway to TORC1 and Cdc25 regulation [43, 44]. Lucena et al. also reported that Cdc25 phosphorylation and dephosphorylation still occur in *pab1*Δ mutants [23]. This study did not address however, the role of Srk1- dependent Cdc25 phosphorylation during the normal cell cycle (reviewed below). As Srk1- dependent phosphorylation of Cdc25 does not involve the phosphorylation of Cdc2 consensus sites, sequential and differential phosphorylation or combinations thereof may determine the precise timing of mitosis [23, 25, 45]. TORC1 thus regulates the timing of mitosis by modulating PP2A^Pab1^ activity to inhibit Cdc25 and activate Wee1. In contrast, TORC1 inhibition results in Cdc25 activation and the degradation of Wee1 [18, 19, 44]. As PP2A^Pab1^ and Clp1 also regulate the phosphorylation, activity and localisation of Wee1, these pathways serve to integrate Cdc25 and Wee1 activity for the proper timing of mitosis [18, 19, 30].

### DNA damage checkpoints and Cdc25 inhibition

Stalled replication during S- phase or DNA strand breaks in G2, activate the Rad3 regulated DNA damage response pathway and respective downstream activation of Cds1 and Chk1 kinases (reviewed in [3, 4]). The Cds1 and Chk1 kinases in turn, phosphorylate key inhibitory serine and threonine residues on Cdc25. In addition to inhibiting Cdc25 activity within the nucleus, the phosphorylation of these residues also facilitates binding of the 14-3-3 protein Rad24, nuclear export and sequestration within the cytoplasm [46–49]. Interestingly, Cdc25 levels accumulate in the cytoplasm under conditions of cell cycle arrest following DNA damage. Cdc25 levels also accumulate when cell cycle mutants cease dividing at the restrictive temperature. This “stockpiling” of inactive Cdc25 may facilitate rapid cell cycle re-entry following the completion of DNA damage repair [31]. Later studies indicated that Cdc25 nuclear export is not required for DNA damage checkpoint enforcement, indicating that Cds1 or Chk1 mediated phosphorylation is sufficient to inhibit the activity of the phosphatase [47, 50].

Other studies suggest that additional redundant pathways exist, for the regulation of Cdc25 mutants that cannot be phosphorylated [51, 52]. When the 9- 12 major inhibitory phosphorylation sites are mutated (Cdc25_(9A_)-GFP_int_, Cdc25_(12A)_-GFP_int_), *S. pombe* cells are still able to activate an effective DNA damage response. This form of DNA damage checkpoint activation, results from the rapid degradation of these mutant Cdc25 isoforms and a Mik1 dependent cell cycle arrest [51, 52]. The Cdc25_(9A)_-GFP_int_ and Cdc25_(12A)_-GFP_int_ expression levels are relatively stable under normal cell cycle conditions, accumulate in the nucleus to a greater extent than the wild type Cdc25 -GFP_int_ but have a slightly shorter half- life.

Enforced nuclear localisation of Cdc25 (Cdc25- NLS- GFP_int_) does not affect replication checkpoint activation and stockpiling of the phosphatase occurs as with the wild type isoform. The levels of Cdc25- NLS- GFP_int_ are also relatively higher, than in wild type Cdc25- GFP. In contrast, Cdc25_(9A)_- NLS- GFP_int_ is degraded when the replication checkpoint is activated. Cdc25_(9A)_- NLS- GFP_int_ also appears to be relatively unstable compared to Cdc25- NLS- GFP_int_, suggesting Cdc25 phosphorylation prevents degradation during the normal cell cycle [51, 52]. These observations indicate that Cdc25 degradation occurs in the nucleus following stalled replication or DNA damage. They also suggest that activation of the replication or DNA damage checkpoints, induces an increase in the rate of non- phosphorylated Cdc25 degradation. In this regard, it is important to note that Cut8 localises the 26S proteasome to the nucleus, accumulates following DNA damage and is required for DNA repair. However, mutants lacking *cut8*^+^ are checkpoint proficient [53]. As wild type Cdc25 degradation is not required for replication stress or DNA damage- induced cell cycle arrest, it would be interesting to study the impact of a *cut8* deletion on Cdc25_(9A)_-GFP_int_ and Cdc25_(12A)_-GFP_int_ degradation. Cds1 or Chk1- mediated phosphorylation of the major inhibitory phosphorylation sites is thus sufficient to prevent degradation by the 26S proteasome. Other lines of evidence suggest, that the Rad3 regulated checkpoint pathways regulate Cdc25 expression and stability even under normal growth conditions. Deletion of *rad3*^+^ or *cds1*^+^ suppressed *cdc25*^+^ mRNA expression but induced the accumulation the Cdc25 protein. Unlike wild type cells, *rad3*Δ mutants continue to express Cdc25 even in stationary phase [21]. Similarly, the rate of degradation of Cdc25_(9A)_-GFP_int_ and Cdc25_(12A)_-GFP_int_ mutant protein is delayed in a *cds1*Δ background [52]. Rad3 may thus regulate Cdc25 stability in a Cds1- dependent manner even under normal growth conditions. Cds1 also accumulates in response to TORC1 inhibition following glucose withdrawal, providing a further link between TORC and DNA damage checkpoint signalling [54]. While the Pub1 E3- ligase targets Cdc25 to the 26S proteasome for degradation, deletion of *pub1*^+^ did not prevent the degradation of Cdc25_(9A)_-GFP_int_ mutant protein in the presence of hydroxyurea [52]. Furthermore, *pub1*Δ mutants have elevated Cdc25 levels, fail to adequately degrade the phosphatase at mitosis and are sensitive to genotoxic agents [23, 32, 55]. Interestingly, mutants also display sensitivity to caffeine ([56], Alao and Sunnerhagen, unpublished results). It is thus possible that the APC mediates the degradation of Cdc25 isoforms lacking major inhibitory phosphorylation sites, following the activation of the replication or DNA damage checkpoints [39, 40, 52]. Indeed, APC mediated degradation of mitotic cyclins and regulators is required for proper exit from mitosis and progression through cytokinesis [39]. Clp1 is also required for full activation of Cds1 in response to replication stress [57]. Interactions between the replication checkpoint and spindle checkpoint pathways also contribute to the enforcement of cell cycle arrest under genotoxic conditions. These interactions may also contribute to the regulation of Cdc25 stability, via differential combinations of positive (Cdk1, Plo1 mediated) and negative (Cds1, Chk1, Srk1 mediated) phosphorylation of serine/threonine residues [3, 21, 29, 40, 58] in DNA damage checkpoints and Cdc25 inhibition section [59–65].

In Cdc25_(9A)_-GFP_int_ mutants Mik1 is required for effective maintenance of the replication checkpoint [51, 52]. Thus, while Rad24 binding slightly enhances Cdc25 stability under normal growth conditions, it prevents the degradation of the phosphatase when the DNA damage or replication checkpoint pathways are activated. The existence of these redundant mechanisms suggests that even modest Cdc25 activity during DNA damage checkpoint activation can contribute to inappropriate progression through mitosis [21, 51, 52].

Genomic studies have also revealed a role for the DNA damage response pathway, in mediating resistance to caffeine. Mutants with *rad3*Δ, *rad51*Δ, or *rad54*Δ mutations also show sensitivity when grown on solid media in the presence of caffeine [22]. Caffeine may thus induce DNA damage, but the underlying mechanisms remain unclear. It is interesting to note however, that these findings hint at caffeine- induced DNA damage and Rad3 activation in *S. pombe*. Caffeine also appears to accelerate the timing of mitosis under genotoxic conditions, rather than delaying cell cycle progression. Together, these observations provide additional evidence that Rad3 is in fact not a target of caffeine in this organism [21].

### Effect of caffeine on Cdc25 expression and stability

Caffeine can inhibit several members of the PIKK family, and inhibition of Rad3 and its homologues ATM and ATR was thought to be the mechanism underlying checkpoint override [10, 12–14]. This paradigm has proved controversial, as checkpoint override by caffeine can occur in the absence of ATM, ATR or Rad3 inhibition [15, 21, 66]. It has also become apparent, that TORC1 and not ATM homologues are the preferred target of caffeine in vivo [15–17]. TORC1 regulates the timing of cell division in response to nutrient availability via the *S. pombe* Greatwall kinase homologue Ppk18 [18, 67]. Inhibition of TORC1 activity activates Cdc25, induces Wee1 degradation and advances cells into mitosis. The exposure of *S. pombe* cells to caffeine advances mitosis in a manner that resembles TORC1 inhibition [21]. Caffeine also moderately activates the Sty1- regulated ESR pathway [21, 22]. Modest Sty1 activation can drive cells into mitosis in a manner dependent on Plo1 and Cdc25 [6, 43]. Activation of Sty1 has been shown to induce Cdc25 stabilisation, presumably as a consequence of Srk1- mediated phosphorylation, Rad24 binding and sequestration within the cytoplasm [25]. Caffeine may thus modulate cell cycle progression by partially inhibiting TORC1, moderately activating Sty1 or otherwise modulating Cdc25 activity to advance mitosis. In fact, Cdc25 expression was necessary for caffeine- mediated DNA damage checkpoint override in our studies [21]. Previous studies have shown that caffeine induces the accumulation of Cdc25B in mammalian cells [20]. We have similarly demonstrated that caffeine induces the accumulation of Cdc25 in *S. pombe* under normal cell cycle conditions as well as under environmental stress or genotoxic conditions. This effect on Cdc25 occurs at the post-translational level since caffeine suppresses *cdc25*^+^ mRNA expression. Interestingly, *rad3∆* and *cds1∆* deletions also stabilised Cdc25 protein levels while supressing its mRNA expression. Furthermore, caffeine is more effective at advancing mitosis in *rad3*Δ and *cds1Δ* mutants relative to wild type cells [21]. We also noted that DNA damage checkpoint mutants do not just fail to arrest cell division but are accelerated into mitosis following DNA damage. This change in cell cycle kinetics resembles the effect of caffeine on cells exposed to genotoxic agents [21, 29, 68, 69]. Caffeine thus mimics the loss of DNA damage checkpoint signalling in *S. pombe*, without inhibiting Rad3 activity [21]. This effect of caffeine also mimics that of the Tor2 inhibitors rapamycin and torin1 on cell cycle progression in *S. pombe* [44]. Mutants lacking functional Clp1 or Srk1 that normally negatively regulate Cdc25 are more sensitive to caffeine mediated DNA damage checkpoint override than wild type cells. The phosphorylation of Cdc25 is therefore not required for the stabilising effect of caffeine on the phosphatase but influences its effect on cell cycle progression [21].

Caffeine inhibits the degradation of Cdc25 mutants (Cdc25_(9A)_-GFP_int_ and Cdc25_(12A)_-GFP_int_) lacking the major inhibitory phosphorylation sites [21, 51, 52]. In contrast to the stockpiling of wild type Cdc25 when cells are arrested, the Cdc25_(9A)_-GFP_int_ and Cdc25_(12A)_-GFP_int_ mutants are degraded in the presence of genotoxic agents. Redundant mechanisms thus exist, to clear excess non- phosphorylated Cdc25 from the nucleus when DNA damage checkpoint signalling is activated [51, 52]. Caffeine clearly stabilised these mutants in the presence of genotoxic agents [21]. As Cdc25_(9A)_-GFP_int_ and Cdc25_(12A)_-GFP_int_ are relatively stable under normal cell cycle conditions, caffeine must inhibit a pathway that targets non- phosphorylated Cdc25 for ubiquitin- dependent 26S proteasomal degradation under genotoxic conditions. The ability of caffeine to override checkpoint signalling in cells expressing these mutants, is also enhanced relative to the wild type protein [21, 51, 52].

The rapid degradation of Cdc25 isoforms that cannot be phosphorylated (Cdc25_(9A)_-GFP_int_ and Cdc25_(12A)_-GFP_int_) [51] following genotoxic insults, hints at an increase in 26S proteasome mediated protein degradation. This redundant mechanism clears Cdc25 that is unphosphorylated from the nucleus [51, 52]. These studies also demonstrated that Cdc25 protection from degradation occurs via Chk1 and Cds1 inhibitory phosphorylation. As these isoforms are relatively stable under normal cell cycle conditions, genotoxic conditions must somehow enhance the targeting of unphosphorylated Cdc25 to the 26S proteasome [51, 52]. Caffeine thus suppresses Cdc25 degradation independently of Cds1, Chk1 and Srk1- mediated phosphorylation [21]. In fact, exposure to 0.6 M KCl induced the degradation of Cdc25_(9A)_-GFP_int_ in a manner similar to what was observed with genotoxic agents (Alao and Sunnerhagen, unpublished results).

### Mechanisms underlying caffeine- induced Cdc25 stabilisation

By what mechanism(s) could caffeine affect the rate of Cdc25 degradation via the 26S proteasome? Caffeine has been reported to induce the ubiquitin- dependent degradation of certain proteins in mammalian cells [70]. The rapid degradation of Cdc25 isoforms that cannot be phosphorylated under genotoxic conditions, hints at the activation (or increased activity) of a ubiquitin- dependent degradation pathway. Alternatively, a general increase in the overall rate of ubiquitin- dependent degradation may occur under these conditions. Clearly further studies on the regulation of Pub1 (the E3- ligase targeting Cdc25) activity under normal and genotoxic conditions, in the presence and absence of caffeine are warranted. Such studies may also provide novel insights into the regulation of Cdc25 stability in *S. pombe*. Similarly, Cut8 is required to localise the 26S proteasome to the nucleus and plays an important role in DNA damage repair. Cut8 accumulates in response to DNA damage but is not required for checkpoint activation [53, 71]. The accumulation of Cut8 in the presence of genotoxic agents suggests a possible increase in the levels of ubiquitin- dependent protein degradation and could also drive progression through mitosis. Inhibiting Cut8 accumulation could be one possible mechanism, whereby caffeine attenuates the ubiquitin- degradation of nuclear Cdc25 (Alao and Sunnerhagen, unpublished results). Interestingly, *cut8*Δ mutants also display sensitivity to caffeine [22]. These observations suggest that caffeine is itself a DNA damaging agent [22, 53] and may complicate studies on the effect of the drug on the DNA damage response pathway. Nevertheless, the ability of caffeine to override checkpoint signalling and drive cells trough mitosis appears to underlie its chemo- and radio- sensitising effects [9]. Lastly, studies on the effect of caffeine- mediated TORC1 inhibition in the context of mitotic progression are also potentially important. TORC1 mediates the timing of mitosis, by co-ordinating the phosphorylation, activity and expression levels of Cdc25 and Wee1 [18, 23, 44]. The effect of caffeine on cell cycle progression resembles that of more typical TORC1 inhibitors by accelerating the timing of mitosis in *S. pombe* [21, 44]. Caffeine could thus advance the timing of mitosis, by indirectly increasing Cdc25 activity while inhibiting the activity of Wee1. Comparing the effects of TORC1 inhibitors on checkpoint activation with those of caffeine would be interesting. New antibodies that detect hyperphosphorylated Cdc25 and Wee1 have recently been reported. Studies on the effect of caffeine on cell cycle progression in various genetic backgrounds (e.g. mutants of the TORC1 signalling pathway such as *pab1*Δ) using these tools would also be useful [23].

## Conclusion

Despite more than two decades of research, the precise mechanisms whereby caffeine overrides checkpoint signalling remain unclear [9, 10, 17, 21, 66]. The more recent findings that TORC1 and not Rad3 appears to be the major target of caffeine in vivo, is particularly relevant in this regard [15]. It is thus likely that caffeine override DNA damage checkpoint signalling independently of Rad3 inhibition. Modulation of TORC activity by caffeine could account for its effects on cell cycle progression [17, 44] (Fig. 1). Furthermore, caffeine also targets other pathways, at least some of which interact with each other [21, 29]. Clearly, understanding how caffeine suppresses the degradation of Cdc25 in *S. pombe* is of central importance. Studies of this nature may shed light not only on the molecular pharmacology of caffeine, but also on how signalling pathway crosstalk impacts on cell cycle regulation. With the new insights and tools available, we can look forward to many more years of exciting research in this area.

## Data Availability

Not applicable.

## Abbreviations

APC: AnaphasePromoting Complex
ATM: Ataxia telangiectasia mutated
ATR: Ataxia and rad related (ATR) kinase homologue Rad3
DDR: DNA damage response
ESR: Environmental stress response
PIKK: Phosphatidylinositol 3 kinase-like kinase
TORC1: Target of rapamycin complex 1
